# Analysis of Bilaterality and Symmetry of Posterior Staphyloma in High Myopia

**DOI:** 10.3390/diagnostics13162680

**Published:** 2023-08-15

**Authors:** José M. Ruiz-Moreno, Mariluz Puertas, Ignacio Flores-Moreno, Elena Almazán-Alonso, María García-Zamora, Jorge Ruiz-Medrano

**Affiliations:** 1 Department of Ophthalmology, Puerta de Hierro-Majadahonda University Hospital, 28222 Madrid, Spain; 2 Department of Ophthalmology, Castilla La Mancha University, 02001 Albacete, Spain; 3 Miranza Corporation, 28004 Madrid, Spain; 4 Clínica Suárez Leoz, 28010 Madrid, Spain

**Keywords:** bilaterality, high myopia, pathologic myopia, posterior staphyloma, symmetry

## Abstract

The purpose of this study was to examine bilaterality and symmetry of posterior staphyloma (PS) in high myopic eyes. **Methods:** This cross-sectional and non-interventional study assessed 473 high myopic eyes [axial length (AL) ≥ 26 mm] of 259 patients. Patients underwent an ophthalmological examination including multimodal-imaging and myopic maculopathy grading according to Atrophic/Tractional/Neovascular (ATN) system, presence and subtype of PS, and severe pathologic myopia (PM). Bilaterality of PS and subtype’s symmetry between eyes of the same patient was assessed. Four groups were analyzed: (1) bilateral vs. unilateral PS’s eyes. Within bilateral group, symmetric vs. asymmetric subtypes according to (2) Curtin’s classification, (3) Ohno-Matsui’s classification, and (4) primary/compound subtypes. **Results:** Out of the total, 334 myopic eyes of 167 patients were included. The 92.8% (*n* = 310/334) of the eyes presented PS and was bilateral in 85.6% (*n* = 143/167) of the patients. Bilateral eyes showed significantly (*p* < 0.01) greater AL, severe PM, A and N components vs. unilateral PS. AL-difference between both eyes was greater in unilateral PS (*p* < 0.01). Among bilateral PS, the subtype was symmetric in 79 (55.2%), 84 (58.7%), and 115 (80.4%) patients according to Curtin’s classification, Ohno-Matsui’s classification, and primary/compound; respectively. The asymmetric group presented worse best-corrected visual acuity (*p* < 0.01), higher AL (*p* < 0.01), incidence of PM, and severe PM (*p* < 0.05). **Conclusions:** PS was bilateral in most of the patients without clinical differences between both eyes, being symmetrical in more than half of bilateral cases. Patients with bilateral PS showed higher myopic maculopathy, AL, and incidence of severe PM than unilateral PS.

## 1. Introduction

Posterior staphyloma (PS) was defined as a localized outpouching of the ocular wall with a radius of curvature less than the surrounding curvature of the ocular wall [1], and it should be differentiated from simple scleral backward bowing and from secondary acquired-staphylomas [2].

PS was initially described by Scarpa and Briggs in 1801 [3] and has been the subject of multiple investigations over the years. Curtin [4] was the first to propose a PS’s classification using binocular indirect ophthalmoscopy, classifying 10 different subtypes—five primaries and five compounds. Recently, the development of imaging techniques has made it possible to study highly myopic eyes with different diagnostic methods. Using 3D magnetic resonance imaging (MRI), Moriyama et al. [5] analyzed the entire shape of eyes with pathologic myopia (PM) and, thus, was able to visualize large PS. Subsequently Ohno-Matsui [6], using 3D-MRI and wide-field fundus imaging, proposed classifying PS into six subtypes—based on Curtin’s classification but renamed according to the location of the outpouching of the ocular globe.

Although PS is considered a hallmark of PM [7,8,9,10,11], it is not pathognomonic, since other pathologies could also develop PS [12,13,14]. Regardless, it has been included as one of the defining characteristics of PM according to the definition of the META-PM group [7,15]. Ruiz-Moreno et al. [16] have recently confirmed that PS was associated with myopic maculopathy and worse visual function. Becoming, therefore, a determining factor for myopic maculopathy, which is associated with more severe myopia-related disorders compared to non-PS’s eyes [5,6,8,9,11,17,18].

It has been previously described that increasing AL and age, following this order [16], constitute the main factors associated with the development of PS [10,19,20]. However, the underlying pathogenesis of PS remains unclear. The reason whereby, despite common clinical features, some high myopic eyes develop PS, while others do not, is currently being investigated.

The potential heritability of high myopia has raised controversy among investigators, the presence of genetic differences between high myopia and PM has not been fully elucidated. It has been suggested that the development of myopic maculopathy might be partially influenced by genetics, which also applies for the onset of PS [10].

The aim of this study was to analyze the bilaterality of PS in high myopic eyes, as well as the symmetry between the subtype presented among patients with bilateral involvement.

## 2. Materials & Methods

This was a cross-sectional and non-interventional study of 473 consecutive high myopic eyes of 259 patients conducted at Puerta de Hierro-Majadahonda University Hospital (Madrid, Spain), tertiary referral hospital for vitreoretinal pathology. The procedures and the study design adhered to the tenets of the Declaration of Helsinki and approval was obtained from the corresponding Institution’ Ethics Committee (PI 43/20). All subjects had signed an informed consent form and were identified through a medical record search during the period from June 2021 to December 2022.

Inclusion criteria were the presence of high myopia [defined as axial length (AL) ≥ 26 mm] in both eyes, presence of PS in at least one of the eye (although both eyes had to be diagnosed with high myopia, only one of them had posterior staphyloma), age ≥ 18 years, and availability of good quality ocular images [>45 image quality score] on the DRI Triton Swept-Source (SS) optical coherence tomography (OCT) software 1.02.1 (Topcon Corporation, Tokyo, Japan).

The exclusion criteria included previous intraocular surgery (except for refractive, cataract surgery, or vitreoretinal surgeries secondary to high myopia—traction myopic maculopathy), history of vitreoretinal or glaucoma surgeries that could have affected the shape of the globe, and/or coexisting ocular or systemic diseases including optic nerve pathology.

**Figure 1:**
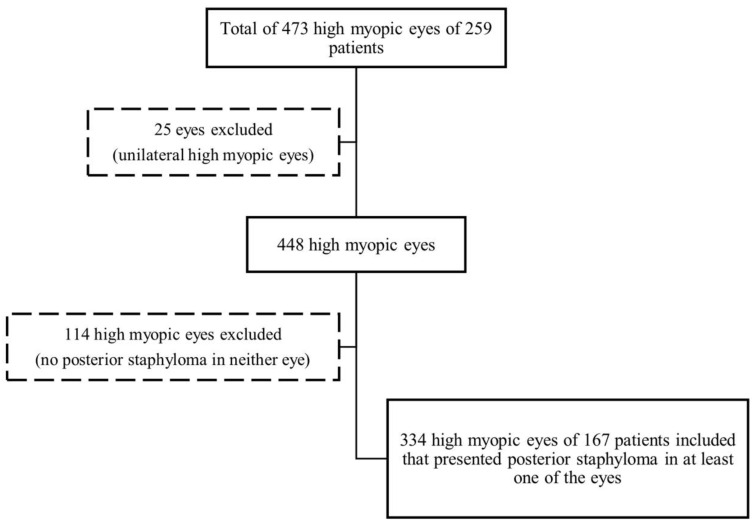
Flow diagram of the patients/eyes’ selection process.

All examinations were performed in both eyes. If both eyes fulfilled the study’s inclusion/exclusion criteria, the images were independently provided. (Flow Diagram, Figure 1).

Demographic data were obtained from the patients’ clinical records. All participants underwent a complete ophthalmological examination that included: best corrected visual acuity (BCVA) measured in decimal, slit-lamp anterior segment examination, intraocular pressure (Goldman applanation tonometry), stereoscopic fundus examination by indirect ophthalmoscopy under mydriasis, and AL measurement with optical biometer (IOL Master 500, Carl Zeiss Meditec AG, Jena, Germany).

### 2.1. Multimodal Imaging

Different imaging examinations were performed in all subjects. Multimodal imaging included color fundus photography using Zeiss Clarus TM 500 (Carl Zeiss Meditec AG, Jena, Germany) and/or DRI-OCT Triton^®^ plus (Topcon Corporation, Tokyo, Japan). SS-OCT, OCT-angiography obtained with DRI-OCT Triton plus, and fundus autofluorescence with Spectralis^®^ OCT (Heidelberg Co., Heidelberg, Germany) were also performed. Structural OCT protocol included cross-sectional and 12-mm radial scans centered on the fovea containing each 1024 axial scans. Fluorescein angiography or OCT-angiography were performed if myopic choroidal neovascularization (CNV) was suspected.

### 2.2. ATN Grading System

The ATN grading system [21,22] for myopic maculopathy was blinded evaluated by two retinal specialists that assessed the atrophic (A), tractional (T), and neovascular (N) components based on fundus photography and SS-OCT scans. Regarding T component, in those patients who had undergone vitreoretinal surgery due to tractional maculopathy, the traction considered was the presurgical T score avoiding bias caused by post-surgical outcome. In addition, other conditions taken into account were that eyes with myopic CNV or myopic full-thickness macular hole were included in the study as long as the atrophic component was equal to or higher than diffuse atrophic maculopathy (A2). These criteria were applied to prevent including eyes with CNV or full-thickness macular hole secondary to other pathologies.

### 2.3. Pathologic Myopia and Severe Pathologic Myopia

Eyes were analyzed if they met the criteria of PM or severe PM based on ATN grading system. PM was defined as ≥A2 according to META-PM definition [7] and severe PM as ≥A3 (patchy atrophy), ≥T3 (foveal detachment), and/or N2 (active/scar myopic CNV or Fuchs spot) [23].

### 2.4. Posterior Staphyloma

The presence and subtype of PS were determined by indirect ophthalmoscopy, fundus photography, and OCT by two blinded retinal specialists (IF-M and MP). In those eyes without agreement, a third blinded senior consultant retinal specialist (JMR-M) checked and classified the staphyloma. PS were classified according to two classifications:

1. Curtin’s classification [4]: 10 types—five primary and five compound forms— based on their funduscopic appearance according to the area involved.
2. The recent classification defined by Ohno-Matsui [6]: 6 types renamed according to their location and distribution. The first five types coincide with the same as Curtin’s classification and the sixth and last type named “others” where all the compound forms of staphylomas are grouped.

The current study compared and analyzed both classifications. Additionally, two groups were made according to those primaries and compounds posterior staphylomas as described by Curtin [4]. The bilaterality of PS and the subtype’s symmetry between eyes of the same subject were assessed. Initially, it was studied whether the patient presented high myopia in both eyes, excluding those patients with only one high myopic eye, and thus eliminating possible causes of unilateral high myopia. Among the patients with bilateral high myopia, the presence of unilateral or bilateral PS was determined. In those cases with bilateral PS, the concordance between subtypes of both eyes was determined according to Curtin’s [4] and Ohno-Matsui’s [6] classifications, and the symmetry between eyes was also evaluated basing on whether they were primary or compound subtypes.

In summary, different groups were performed and studied: (1) bilateral versus (vs.) unilateral PS’s eyes. Within bilateral PS’s eyes the symmetry between both eyes was analyzed according three different classifications; (2) symmetric vs. asymmetric PS’s subtypes according to Curtin’s classification [4]; (3) according to Ohno-Matsui’s classification [6]; and (4) Symmetric vs. asymmetric eyes regarding if they were both primary/compound subtypes according to Curtin’s classification.

### 2.5. Statistical Analysis

All the statistical analyses were performed with IBM-SPSS statistical software program (IBM-SPSS, v. 28.0.1.0, Chicago, IL, USA). A two-tailed *p*-value < 0.05 was considered statistically significant. Descriptive statistics were provided for normally distributed variables as means with standard deviation (SD) for quantitative variable and *n* (percentage) for categorical variables. The Kolmogorov-Smirnov test was performed to assess the normality or non-normality of the variables.

Categorical variables were compared using Chi-square test for normally distributed and Fisher’s exact test for the non-parametric variables. Continuous normally distributed variables were compared using independent T-Student. Kruskal-Wallis test was used to compare ordinal variables between groups: A, T, and N components. Age, BCVA, AL, AL-difference between right and left eye of the same subject, ATN classification for myopic maculopathy, PM, and severe PM were compared between groups.

Interobserver agreement for ATN grading system and PS’s classifications were analyzed. Concordance correlation coefficient was performed, rho coefficient was considered moderate (≥0.4), good (≥0.6), or excellent (≥0.8) [24,25].

## 3. Results

A total of 473 consecutive high myopic eyes of 259 patients were assessed. Among them, 25 eyes were excluded as they were unilateral high myopic eyes or only one of the eyes could be analyzed with adequate image quality. Among the 448 eyes with bilateral high myopia and enough quality of ocular imaging tests, 114 eyes did not have PS in any eye. Therefore, 334 high myopic eyes of 167 patients were finally included in this study.

Out of the total of eyes, 70.6% were of female patients (*n* = 236/334). The mean age was 65.8 ± 11.3 years (range from 39 to 92 years), mean BCVA was 0.51 ± 0.33 decimal (range from 0.001 to 1), and the mean AL was 30.02 ± 2.59 mm (range from 26 to 37.6 mm). Among the overall eyes, 309 (92.5%) were classified as PM and 187 (56%) met criteria for severe PM.

The mean ATN components were 2.50 ± 0.78, 0.86 ± 1.04, and 0.58 ± 0.88; respectively. Concordance correlation between observers in ATN grading system was excellent, rho coefficients were as follows A: 0.98 (*p* < 0.01); T: 0.98 (*p* < 0.01); and N: 0.99 (*p* < 0.01).

PS was present in 310 (92.8%) eyes. According to Curtin’s classification, 225 (72.6%) were categorized as primaries and 85 (27.4%) as compounds (Table 1). The most frequent subtypes were: peripapillary PS (type III) presented in 62 (20%) eyes, followed by inferior staphyloma (type V) observed in 53 (17.1%), then by narrow morphology PS (type II) in 52 (16.8%), and by wide-macular PS (type I) presented in 33 (10.6%) eyes. On the other hand, based on Ohno-Matsui’s classification, the most common subtype was “others” (which includes all the compounds forms classified by Curtin from subtype 6 to 10) presented in 85 (27.4%) eyes, followed by peripapillary subtype in 62 (20%) eyes, and by inferior PS subtype observed in 53 (17.1%) eyes. Concordance correlation between graders was excellent (rho coefficient = 0.987).

**Figure 2:**
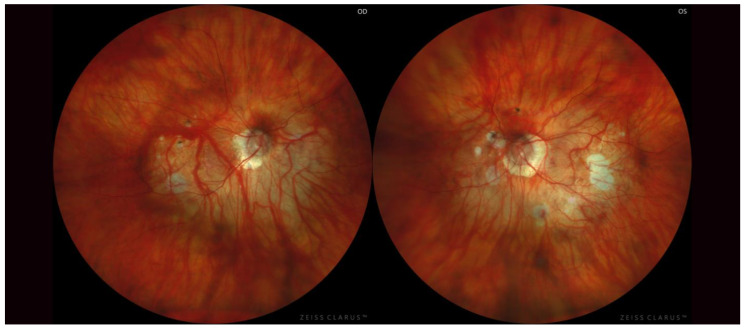
Both highly myopic eyes of the same patient with symmetric posterior staphyloma type I according to Curtin’s classification.

**Figure 3:**
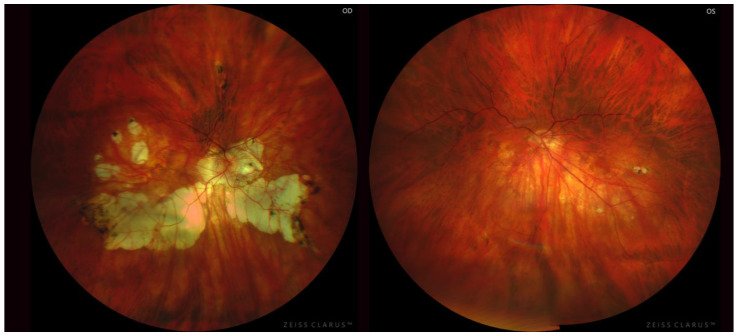
Both highly myopic eyes from the same patient with asymmetric posterior staphyloma. The right eye presented a compound type IX posterior staphyloma—associating a vertical septum on either side of the optic nerve—and the left eye presented a type V posterior staphyloma with the characteristic inferior ectasia.

Regarding bilaterality, 85.6% (*n* = 143/167 patients; *n* = 286/334 eyes) of the patients showed bilateral PS (Figure 2); being unilateral in the 14.4% (*n* = 24/167 patients, *n* = 24/334 eyes) remaining (Figure 3). Eyes with bilateral PS and those with unilateral PS were compared. When the PS was bilateral the AL was significantly greater (*p* < 0.01), as well as A and N components (*p* < 0.01) compared to unilateral PS. Nonetheless, there were not statistically differences (*p* > 0.05) between age, BCVA, and T component. The incidence of severe PM was higher in bilateral PS (*p* < 0.05). On the other hand, the AL-difference between right and left eye of the same patient was greater in patients with unilateral PS (*p* < 0.01) (Table 2).

When comparing both eyes of patients with bilateral PS (right eye vs. left eye) there were no statistically significant differences (*p* > 0.05) regarding age, AL, BCVA, prevalence of PM, or severe PM. The ATN grading in the three components and PS’s subtype according to Curtin’s and to Ohno-Matsui’s classifications were also not significantly different (*p* > 0.05) between both eyes.

Among patients with unilateral PS, both eyes were compared (PS’s eye vs. non- PS’s eye). Eyes with PS showed higher AL (*p* < 0.01), worse BCVA (*p* < 0.05), higher A (*p* < 0.01) and T component (*p* < 0.01), and higher incidence of PM and severe PM (*p* < 0.05). Among patients with bilateral PS, symmetry between PS’s subtype was evaluated. Following Curtin’s classification, the symmetry between the subtype of both eyes was present in 79 (55.2%) patients and according to Ohno-Matsui’s classification the symmetry was found in 84 (58.7%) ones. Additionally, basing on whether the PS was primary or compound, the symmetry between both eyes was 80.4% (*n* = 115/143) (Table 3).

The symmetric and asymmetric eyes were compared. According to the three classifications performed (Curtin’s and Ohno-Matsui’s classifications, and primary/compounds division), the asymmetric group showed worse BCVA, greater AL, and greater A component of myopic maculopathy (*p* < 0.01 each, respectively). On the contrary, not statistically differences were observed (*p* > 0.05) regarding age, AL-difference between right and left eye, and T and N component of myopic maculopathy. Furthermore, asymmetric eyes showed higher incidence of PM and severe PM (*p* < 0.05) than the symmetric ones; these results agreed in the three analyses previous mentioned (Table 4).

## 4. Discussion

Recently, our group has shown that PS was associated with myopic maculopathy, worse visual acuity, and higher prevalence of severe PM. Furthermore, that AL and age, following this order, were the main factors associated with the onset of PS [16]. Other authors had published similar findings, reporting worse BCVA in high myopic eyes that presented PS compared to those without PS [6,9,20,26,27]. The presence of PS was significantly related to myopic maculopathy and poorer visual function [18]. Therefore, it seems to have been demonstrated that PS is the main determinant factor of myopic maculopathy, which in turn is the main cause of vision loss in patients with high myopia [2,6,7,8,9,10,11,16,17,18]. However, surprisingly, there have been no previous studies on its bilaterality and symmetry as it affects a paired organ as are the ocular globes.

Improving the knowledge of PS, its genesis, the characteristics of symmetry, and bilaterality would give us information, either for or against, a possible genetic origin. However, considering that both eyes of the same person have been exposed to the same ambient light stimuli, there would be data supporting the influence of environmental factors on its development [28,29]. The underlying pathogenesis of PS has been not fully understood yet. Current research is still investigating why, despite having common clinical features, some eyes with high myopia develop PS, while others do not. It has been postulated that several different mechanisms (involving the process of emmetropization and structural defects in collagen fibers) may cause posterior staphyloma. The sclera [30,31] has historically been considered the primary tissue involved in the development of posterior staphyloma. However, recent studies have suggested the possibility of prior changes in other structures, such as the choroid [32,33,34] and/or Bruch’s membrane [35,36,37,38,39].

In this study, bilateral PS was found in 85.6% of patients with bilateral high myopia, while only 14.4% showed unilateral PS. There were no statistically significant differences in BCVA or AL between right and left eye of the same patient in the group with bilateral PS. On the contrary, patients with bilateral PS showed significantly greater AL, greater A and N components, and higher incidence of severe PM than those unilateral PS.

These findings would support a genetic background in the development of PS; since these cases had greater the AL, greater myopic maculopathy, and greater incidence of PM and severe PM. These patients with bilateral PS had worse clinical features than those with unilateral PS. Moreover, the underlaying mechanism of these eyes with unilateral affection would not be easily explainable, as both eyes would have been exposed to the same environment and would carry the same genetic load. It might be hypothesized that unilateral cases represent a fruste manifestation of the disease, as occurs in other ocular diseases such as keratoconus. Classically, the reported rates of unilateral keratoconus were as high as 41% [40]. Recently, however, as a result of new imaging techniques and updated diagnostic criteria, the incidence had been reported from 0 to 17% [41,42] finding several form fruste of keratoconus in the fellow eye. Nonetheless, the asymmetry in keratoconus is practically constant for all the topographic parameters analyzed [43,44,45].

Curtin in 1977 already emphasized, as well as in the current study, the importance of PS and the need for accurate stereoscopic fundus evaluation to identify and classify PS, and that PS should be studied from a possible genetic background. Curtin considered that PS could be the result of a congenitally defective sclera acted upon by abiotrophic defects [4].

It has been previously published, that genome-wide association studies (GWAS) had identified more than 100 susceptibility genes for myopia [46,47,48]. However, the underlying genetic basis of PM has not been fully elucidated. For instance, there is little knowledge about whether all subjects with high myopia have the same risk of developing PM or whether the risk of developing PM depends on the patient’s genetic load [27]. Nowadays, the genetic origin of high myopia, the development of myopic maculopathy, and PS constitutes one of the major lines of research [2,10].

In 2018, GWAS analysis of myopic maculopathy performed in a Japanese population identified CCDC102B as a susceptibility gene for myopic maculopathy [49]. The distribution of CCDC102B genotype was significantly different between the 1381 high myopic cases with myopic maculopathy and the 936 high myopic controls without myopic maculopathy. In contrast, CCDC102B was not significantly associated with AL and no association between CCDC102B and myopia was reported in previous GWAS studies. CCDC102B is a susceptibility gene for myopic maculopathy, but not for myopia [2]. The key factor in myopic maculopathy seems to be PS, therefore an investigation of the genes responsibles for PS is needed. Since it has been demonstrated that the three components of ATN are greater in eyes with myopic maculopathy and with PS than in those with myopic maculopathy but without PS [16] Ohno-Matsui proposed to establish the role of CCDC102B in the development of myopic maculopathy, since the genetic basis for high myopia and for the development of myopic maculopathy may be different [2].

The CCDC102B discovery suggests that it may be able to prevent the onset of myopic maculopathy, even after the appearance of high myopia. To control the development of PM in patients with high myopia, further studies are needed to detect more susceptibility genes for myopic maculopathy probably related to PS development. Given that PS was associated with myopic maculopathy in high myopic eyes [16], the identification of genes that are susceptible for PS would contribute to the future control of myopic maculopathy [50]. Combined studies of GWAS with multimodal analysis to classify the presence and morphology of PS would enable us to improve the management of PM.

Furthermore, there is an animal model—the LRP2 Knockout Mouse—that develops PS [51], which also supports the relevance of genetic factors in its appearance. Besides the PS, the mutated knockout mice also developed retinal and sclera thinning with chorioretinal atrophy similar to PM’s eyes. On the other hand, they maintained normal intraocular pressure throughout their lifetime, which is also consistent with the findings of patients with PM. Thus, the LRP2 knockout mouse may be used as an animal model to study the etiology of PM [2,51].

Regarding the symmetry, in the current series, it was present in 55.2% and 58.7% of patients according to Curtin’s and Ohno-Matsui’s classifications, respectively. Hence, the symmetry values were moderate. Notwithstanding, the percentage were significantly greater when considering primaries vs. compounds subtypes reaching 80.4% of symmetry. Asymmetric PS’s eyes showed worse BCVA, higher AL, greater A component of myopic maculopathy, and higher incidence of PM and severe PM than symmetric eyes. These strongly suggest that the asymmetric group may correspond to advanced stages of PM in which the scleral defects become more severe and with larger involvement. Therefore, ectasias tend, in addition to deepening, to develop in new locations without exactly fulfilling any of the subtypes described in the current classifications. Consequently, with a higher tendency towards asymmetry between both eyes. Further objective and accurate studies are needed, including prospective studies of the evolution of PS, using reproducible and less observer-dependent imaging techniques that would enable us to classify these advanced and complex stages of PS.

The widefield-OCT for the identification of PS in high myopia has proven to be quite accurate and would allow us to improve the detection and classification of PS. Widefield-OCT should be considered as a complementary imaging technique in combination with indirect ophthalmoscopic fundus examination to diagnose PS in patients with high myopia. Thus, it could be used to identify eyes with potential myopic maculopathy [18]. Ohno-Matsui considered that the information obtained by Optos imaging system would be necessary to properly classify PS in clinical practice [6,18].

The limitations of our study were as follows. Firstly, the PS’s classification was performed clinically by indirect ophthalmoscopic examination and, regardless of the masked procedure, a certain subjective component is invariably involved. The existence of an objective method could avoid this limitation, but it does not currently exist despite the improvement brought about using widefield-OCT [6,18]. In second place and undoubtedly, the main limitation was the determination of the AL of the high myopic eye. When referring to primaries’ staphylomas subtypes I and II or compound’s staphylomas, the AL is coincident with the greatest length of the eyeball and therefore constitutes a perfect biomarker, so it is possible to refer as foveal AL, since it is the foveolar fixation that determines the measurement of the AL. However, in the other subtypes of staphylomas (subtypes III, IV and V), there is no reliable objective way of determining the greatest length of the ocular globe (non-axial). Possibly MRI could give us the exact measurement of the greatest length of the globe, which may not correspond to the foveal AL in these cases. Therefore, high myopic cases with PS ought to be assessed with this new concept of measurement of “longer eye length” that would coincide with the foveal AL in PS’s subtypes I, II, and compound, but probably not in III, IV, and V subtypes. This fact would further imply the importance of myopic maculopathy since in these cases in which the PS does not affect the macula, we would not detect high degrees of myopic maculopathy due to the PS is outside the macular region.

## 5. Conclusions

In conclusion, PS was bilateral in most of the patients without myopic clinical differences between both eyes, being symmetrical in slightly more than half of them. Patients with bilateral PS showed significantly greater myopic maculopathy than those with unilateral PS. The asymmetric PS’s eyes presented worse BCVA, higher AL, greater A component, and higher incidence of PM and severe PM.

## Figures and Tables

**Table 1:** Prevalence of posterior staphyloma subtype according to Curtin’s classification [4].

Primaries (%, *n*)		Compounds (%, *n*)	
72.6% (225/310)		27.4% (85/310)	
Type I	10.6% (33/310)	Type VI	0.7% (2/310)
Type II	16.8% (52/310)	Type VII	10.3% (32/310)
Type III	20% (62/310)	Type VIII	2.9% (9/310)
Type IV	8.1% (25/310)	Type IX	8.7% (27/310)
Type V	17.1% (53/310)	Type X	4.8% (15/310)

**Table 2:** Analysis of clinical differences between bilateral and unilateral posterior staphyloma’s eyes.

	Bilateral	Unilateral	*p*
***n* (patients, eyes)**	85.6% (*n* = 143/167, *n* = 286/334)	14.4% (*n* = 24/167, *n* = 24/334)	
**Age (years-old)**	66.28 ± 11.49	63.50 ± 9.93	*p* > 0.05 *
**BCVA (decimal)**	0.51 ± 0.33	0.47 ± 0.35	*p* > 0.05 *
**AL (mm)**	30.27 ± 2.58	28.45 ± 2.04	*p* < 0.01 *
**AL-difference (Right eye AL—left eye AL)**	0.21 ± 1.35	1.25 ± 2.05	*p* < 0.01 *
**A component**	2.58 ± 0.74	2.04 ± 0.87	*p* < 0.01 **
**T component**	0.88 ± 1.05	0.77 ± 1.04	*p* > 0.05 **
**N component**	0.63 ± 0.89	0.25 ± 0.67	*p* < 0.01 **
**Severe pathologic myopia**	60.84% (*n* = 174/286)	37.5% (*n* = 9/24)	*p* < 0.05 ***

A: Atrophic, T: Tractional, N: Neovascular. * Student-T test, ** Kruskal-Wallis test, *** Fisher-test.

**Table 3:** Patients with symmetric and asymmetric posterior staphyloma’s subtype according to Curtin’s classification [4], Ohno-Matsui’s classification [6], and simple/compounds subtypes.

	Symmetric	Asymmetric
**Curtin’s classification** **(%, patients)**	55.2% (*n* = 79/143)	44.8% (*n* = 64/143)
**Ohno-Matsui’s classification** **(%, patients)**	58.7% (*n* = 84/143)	41.3% (*n* = 59/143)
**Simple/compound subtypes** **(%, patients)**	80.4% (*n* = 115/143)	19.6% (*n* = 28/143)

**Table 4:** Comparison of clinical data between eyes with symmetric subtype of posterior staphyloma and asymmetric subtype of posterior staphyloma.

		Symmetric Subtype	Asymmetric Subtype	*p*
**N** **(patients/eyes)**	Curtin’s classification	55.2% (*n* = 79/143, *n* = 158/286)	58.7% (*n* = 84/143, *n* = 168/286)	
	Ohno-Matsui’s classification	58.7% (*n* = 84/143, *n* = 168/286)	55.2% (*n* = 79/143, *n* = 158/286)	
	Primary/Compound	80.4% (*n* = 115/143, *n* = 230/286)	19.6% (*n* = 28/143, *n* = 56/286)	
**Age** **(years)**	Curtin’s classification	67.06 ± 11.31	65.31 ± 11.67	*p* > 0.05 *
	Ohno-Matsui’s classification	66.82 ± 11.30	65.51 ± 11.76	
	Primary/Compound	66.66 ± 11.30	64.71 ± 12.21	
**BCVA (decimal)**	Curtin’s classification	0.53 ± 0.31	0.46 ± 0.34	*p* < 0.01 *
	Ohno-Matsui’s classification	0.53 ± 0.31	0.46 ± 0.35	
	Primary/Compound	0.53 ± 0.31	0.46 ± 0.34	
**AL** **(mm)**	Curtin’s classification	29.77 ± 2.66	30.88 ± 2.35	*p* < 0.01 *
	Ohno-Matsui’s classification	29.97 ± 2.77	30.68 ± 2.22	
	Primary/Compound	30.00 ± 2.57	31.35 ± 2.34	
**AL difference** **RE—LE (mm)**	Curtin’s classification	0.09 ± 1.21	0.36 ± 1.49	*p* > 0.05 *
	Ohno-Matsui’s classification	0.16 ± 1.23	0.29 ± 1.49	
	Primary/Compound	0.15 ± 1.36	0.48 ± 1.29	
**A component**	Curtin’s classification	2.45 ± 0.76	2.74 ± 0.60	*p* < 0.01 **
	Ohno-Matsui’s classification	2.47 ± 0.76	2.74 ± 0.69	
	Primary/Compound	2.53 ± 0.76	2.80 ± 0.62	
**T component**	Curtin’s classification	0.87 ± 1.09	0.89 ± 0.99	*p* > 0.05 **
	Ohno-Matsui’s classification	0.89 ± 1.12	0.86 ± 0.94	
	Primary/Compound	0.87 ± 1.06	0.93 ± 1.02	
**N component**	Curtin’s classification	0.61 ± 0.89	0.66 ± 0.91	*p* > 0.05 **
	Ohno-Matsui’s classification	0.61 ± 0.89	0.66 ± 0.91	
	Primary/Compound	0.61 ± 0.89	0.71 ± 0.91	
**PM** **(%, eyes)**	Curtin’s classification	91.14% (*n* = 144/158)	99.22% (*n* = 127/128)	*p* < 0.05 ***
	Ohno-Matsui’s classification	91.67% (*n* = 154/168)	99.15% (*n* = 117/118)	
	Primary/Compound	93.48% (*n* = 215/230)	100% (*n* = 56/56)	
**Severe PM** **(%, eyes)**	Curtin’s classification	56.33.14% (*n* = 89/158)	66.41% (*n* = 85/128)	*p* < 0.05 ***
	Ohno-Matsui’s classification	57.14% (*n* = 96/168)	66.10% (*n* = 78/118)	
	Primary/Compound	57.83% (*n* = 133/230)	73.21% (*n* = 41/56)	

BCVA: Best corrected visual acuity; RE: right eye; LE: Left eye; PM: Pathologic myopia; A: Atrophic; T: Tractional; N: Neovascular. * Student-T test, ** Kruskal-Wallis test, *** Chi-Square test.

## Data Availability

All data generated and/or analyzed during the study are included in this article. Further enquiries could be directed to the corresponding author.
